# Supplementary material for: A habit and working memory model as an alternative account of human reward-based learning
Source: Nat Hum Behav. 2025 Nov 17;10(2):357–69. doi: 10.1038/s41562-025-02340-0 (PMC12932107; doi:10.1038/s41562-025-02340-0)
Supplement: Supplementary file 1 — Supplementary Tables 1–3, Figs. 1–12 and Discussion. [file 41562_2025_2340_MOESM1_ESM.pdf]

nature human behaviour

Supplementary information https://doi.org/10.1038/s41562-025-02340-0

A habit and working memory model as an alternative account of human reward-based learning

In the format provided by the authors and unedited

Contents

Behavior statistics 2
Model comparison 3
Model identifiability 6
Model validation figures 9
RLWMP validation 20
Supplementary Discussion 23

Supplementary References **24**

References **24**

Behavior statistics

Table 1

Effect of number of previous chosen vs. unchosen - across all set/sizes (Main); NS6 only. 2-sided one-sample t-test without adjustment for multiple comparisons.

Dataset	Main p-value	Main t (df)	Main d	Main 95% CI	NS6 p-value	NS6 t (df)	NS6 d
CF12	8.81×10^{-24}	$t(78) = -14.5$	-1.63	$[-1.85, -1.40]$	0.104	$t(78) = -1.64$	-0.18
SZ	1.01×10^{-4}	$t(80) = -4.09$	-0.45	$[-0.68, -0.23]$	0.137	$t(84) = 1.5$	0.16
EEG	5.38×10^{-10}	$t(39) = -8.18$	-1.29	$[-1.61, -0.97]$	0.0271	$t(39) = -2.3$	-0.36
fMRI	4.61×10^{-7}	$t(25) = -6.74$	-1.32	$[-1.73, -0.92]$	0.0514	$t(25) = -2.05$	-0.40
Dev	5.98×10^{-25}	$t(279) = -11.4$	-0.68	$[-0.80, -0.56]$	NA	NA	NA
GL	1.46×10^{-5}	$t(22) = -5.53$	-1.15	$[-1.59, -0.72]$	0.193	$t(25) = -1.34$	-0.26

Table 2

Effect of set-size on chosen-unchosen errors, with mixed effect regression model `DeltaError` $\sim$ `SetSize + (1|ParticipantID)`. *No adjustment for multiple comparisons.*

Data Set	Estimate	(95% CI)	p-value	t-value
CF12	0.15	$(0.12, 0.17)$	9.16×10^{-29}	12.07
SZ	0.14	$(0.11, 0.17)$	1.17×10^{-21}	10.11
EEG	0.09	$(0.06, 0.12)$	8.64×10^{-09}	6.01
fMRI	0.059	$(0.008, 0.110)$	0.02	2.29
Dev	0.058	$(0.037, 0.079)$	9.96×10^{-08}	5.36
GL	0.056	$(0.008, 0.104)$	0.02	2.29

Table 3

Effect of number of previous chosen vs. unchosen in highest set size - late learning only (iteration>5). 2-sided one-sample t-test without adjustment for multiple comparisons.

Dataset	p-value	t (df)	Cohen's d	95% CI
CF12	0.297	$t(75) = -1.05$	−0.12	[−0.35, 0.11]
SZ	2.96×10^{-5}	$t(78) = -4.44$	−0.50	[−0.72, −0.28]
EEG	0.993	$t(39) = -0.00847$	−0.00	[−0.32, 0.32]
fMRI	0.833	$t(24) = -0.213$	−0.04	[−0.46, 0.37]
Dev	3.61×10^{-7}	$t(274) = -5.22$	−0.31	[−0.43, −0.20]
GL	0.933	$t(25) = -0.0845$	−0.02	[−0.42, 0.39]

Model comparison

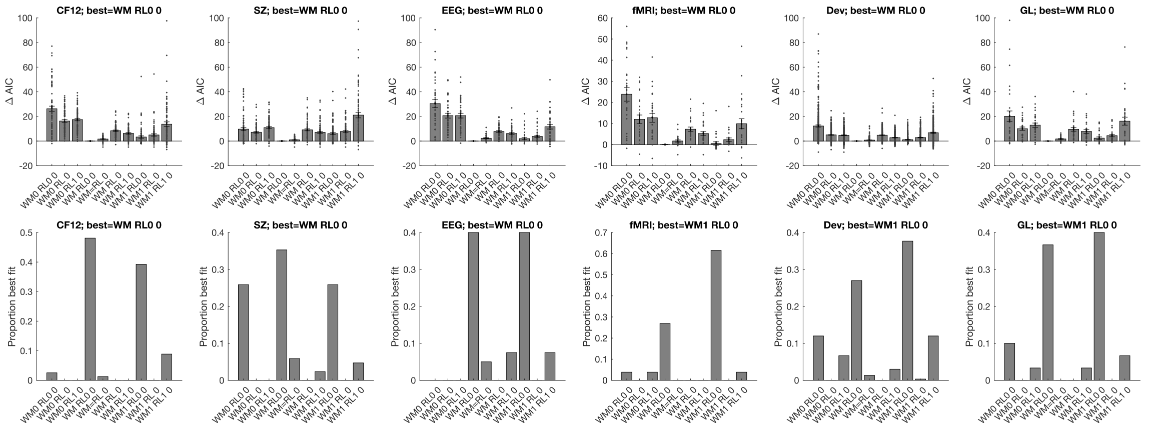

Figure S1

Model comparison within standard RLWM model family. Models are indexed by their modules (WM or RL; see methods); the bias term within their module (0 indicates $\alpha - = 0$; 1 indicates $\alpha_- = \alpha_+$, no number indicates a free parameter; = indicates a shared free parameter). Here, the "0" label at the end indicates fixed $r_0 = 0$. The top row plots mean AIC difference to the model with the best group mean AIC (labeled in each panel title); error bars are standard error of the mean, dots are individual data points. The bottom row plots the proportion of participants best fit by each model, and indicates the winning model on this metric. In all data sets, the winning model is either WM RL0 (including a free negative bias parameter for WM, and no learning from negative outcomes in RL), or WM1 RL0, where there is no negative learning bias in the WM module. The number of data points for each dataset is the same as in Main text figure 1.

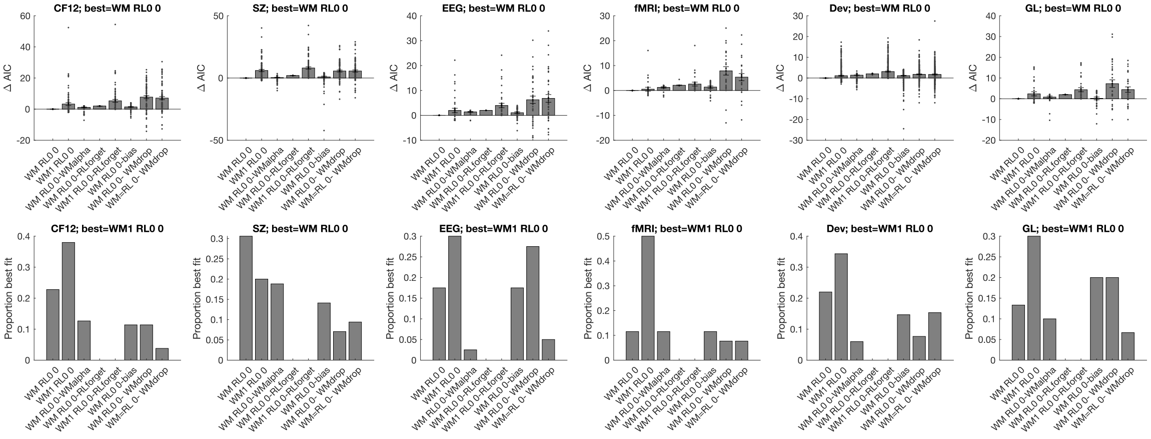

Figure S2

Additional flexibility within RLWM family does not improve fit. The best two models of the previous family (see figure above) are compared to the best models of four additional RLWM families (see methods): a model family with parameter $\alpha_{WM}(1)$ free (labeled here with WMalpha); a model family with a free parameter for forgetting within the RL module (implemented identically to decay in the WM module; labeled here with RLforget); a model family with a choice bias for specific motor actions; (labeled with bias suffix here, see methods); and a model family with no contribution of WM for set sizes above capacity (labeled with WMdrop suffix here). Across data-sets, those models fit significantly worse than models from the standard RLWM family, and consequently, than the WMH models. The top row plots mean AIC difference to the model with the best group mean AIC (labeled in each panel title); error bars are standard error of the mean, dots are individual data points. The bottom row plots the proportion of participants best fit by each model, and indicates the winning model on this metric. The number of data points for each dataset is the same as in Main text figure 1.

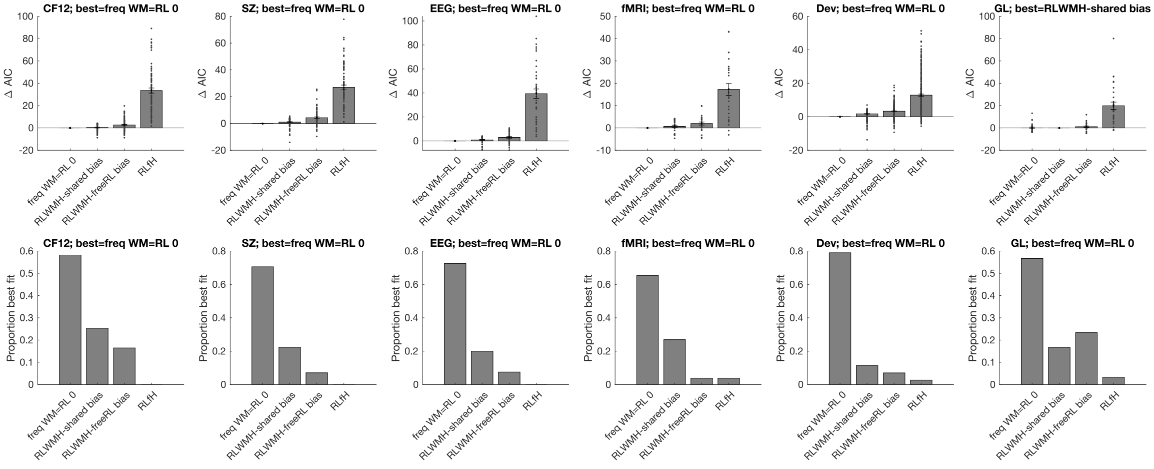

Figure S3

A three factor model does not improve fit. The best overall model (freqWM=RL, the WMH model)) is compared to two other types of models: a model family with that includes three separate modules (WM, H and RL; labeled RLWMH here), with different parameterizations of learning rate bias (see methods); and a model family that combines a forgetful RL model with an H model, without WM (labeled RLfH here). Across all data sets, the winning model from the main text fits best. The top row plots mean AIC difference to the model with the best group mean AIC (labeled in each panel title); error bars are standard error of the mean, dots are individual data points. The bottom row plots the proportion of participants best fit by each model, and indicates the winning model on this metric. The number of data points for each dataset is the same as in Main text figure 1.

Model identifiability

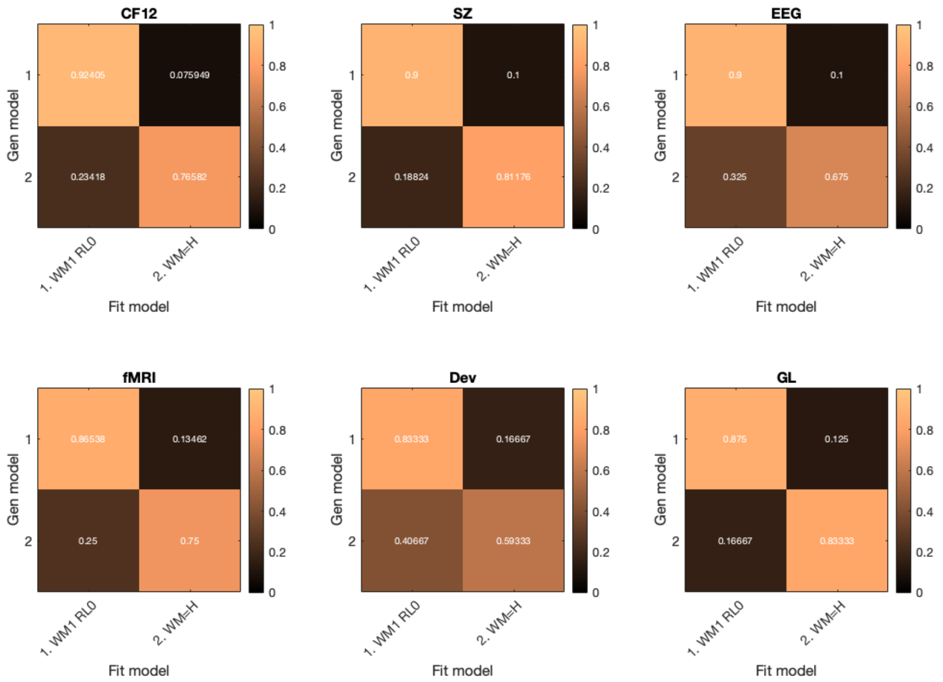

Figure S4

Winning WMH model is identifiable vs. best RLWM model. We simulated artificial datasets using both models and parameters fit on individual participants. For each participants, we simulated enough times to ensure we included at least 100 simulations (e.g., twice per participant in CF12, once in Dev, 4 times in fMRI). We then fit artificial datasets with both models using the same procedure as for real participants. For each dataset, we assign a winning model as the model with the lowest AIC. We verified that BIC overpenalized complexity and lead to worse confusion matrices. WMH is highly recoverable, with the lowest recoverability in the Dev data set where the number of blocks and lack of set-size 6 decreases the difference between H and RL agents.

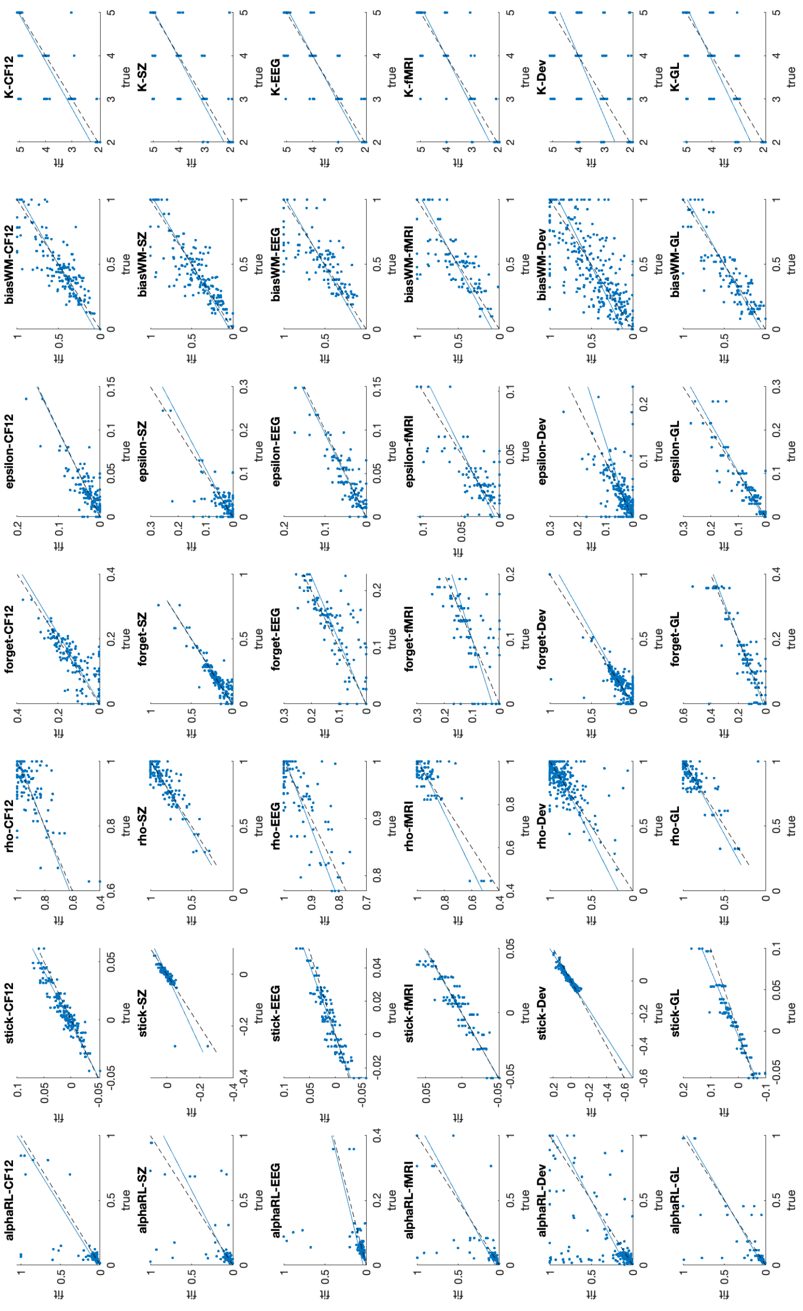
K-CF12
K-SZ
K-EEG
K-fMRI
K-Dev
K-GL
biasWM-CF12
biasWM-SZ
biasWM-EEG
biasWM-fMRI
biasWM-Dev
biasWM-GL
epsilon-CF12
epsilon-SZ
epsilon-EEG
epsilon-fMRI
epsilon-Dev
epsilon-GL
forget-CF12
forget-SZ
forget-EEG
forget-fMRI
forget-Dev
forget-GL
rho-CF12
rho-SZ
rho-EEG
rho-fMRI
rho-Dev
rho-GL
stick-CF12
stick-SZ
stick-EEG
stick-fMRI
stick-Dev
stick-GL
alphaRL-CF12
alphaRL-SZ
alphaRL-EEG
alphaRL-fMRI
alphaRL-Dev
alphaRL-GL
true
fit

Figure S5

*Parameter recovery analysis. Parameters in the winning WMH model are highly recoverable. We simulated and fit the same number of agents as in Supplementary Fig. S4, and compared true generating parameters with fit recovered parameters, obtaining a high correlation for all parameters in all datasets. Dashed black line is unity line; blue line is least squared regression line. Note that this figure also provides the distribution of best fit parameters across the group in all datasets. For visualization purposes, the discrete capacity parameter was slightly jittered with .05*normal noise. All Spearman* $\rho > .56$, $p < 10^{-8}$, *two-sided, uncorrected; results are similar with Pearson correlations. The number of data points for each dataset is the same as in main text figure 1.*

Model validation figures

Following figures include model validation figures for all 6 data sets with the same models as in Main text Figure 2. The data set is indicated in the title of the top row of each figure.

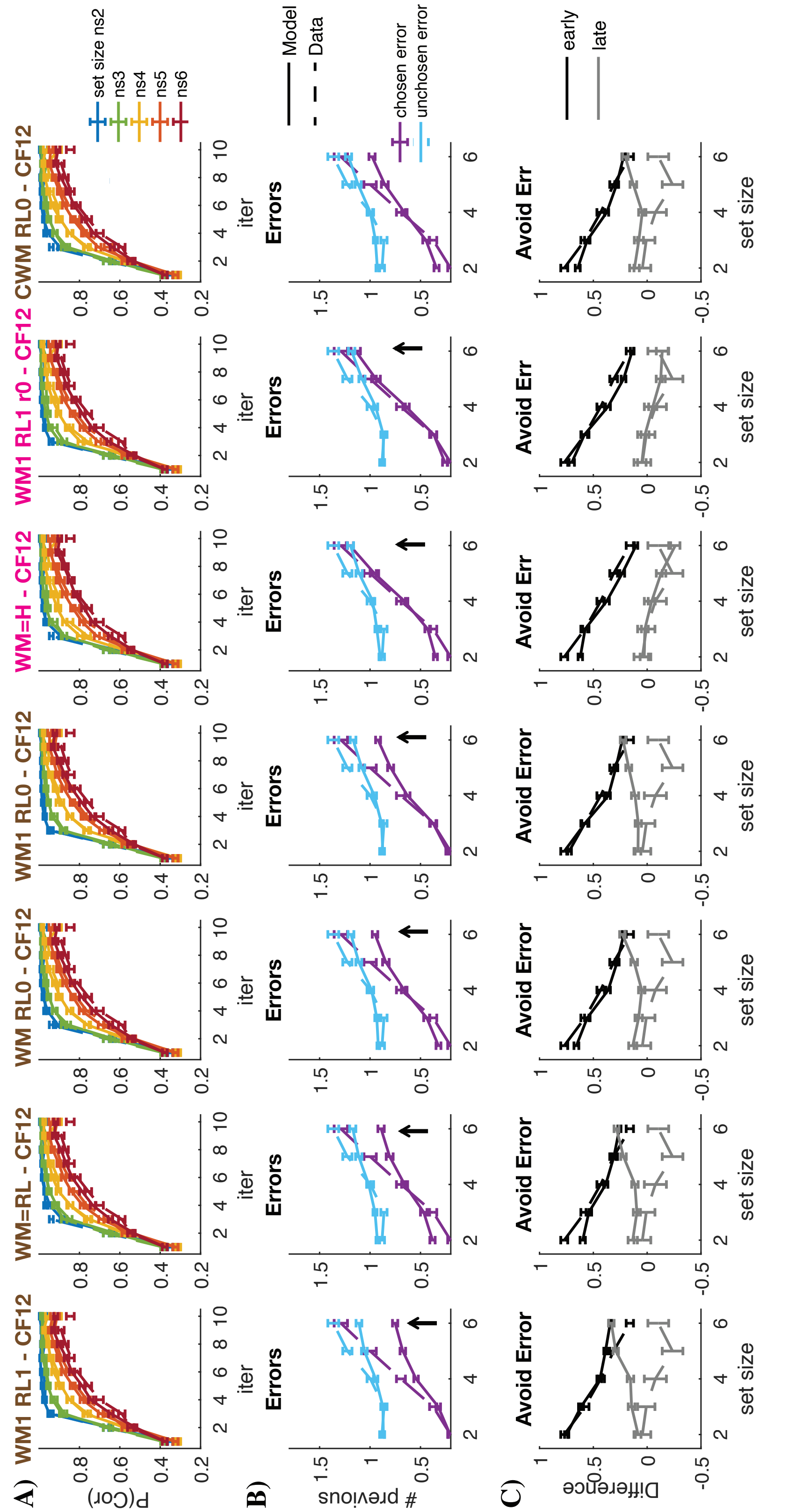
A)
B)
C)
WM1 RL1 - CF12
WM=RL - CF12
WM RL0 - CF12
WM1 RL0 - CF12
WM=H - CF12
WM1 RL1 r0 - CF12
CWM RL0 - CF12
P(Cor)
iter
Errors
previous
Avoid Error
Avoid Err
Difference
set size
set size ns2
ns3
ns4
ns5
ns6
Model
Data
chosen error
unchosen error
early
late

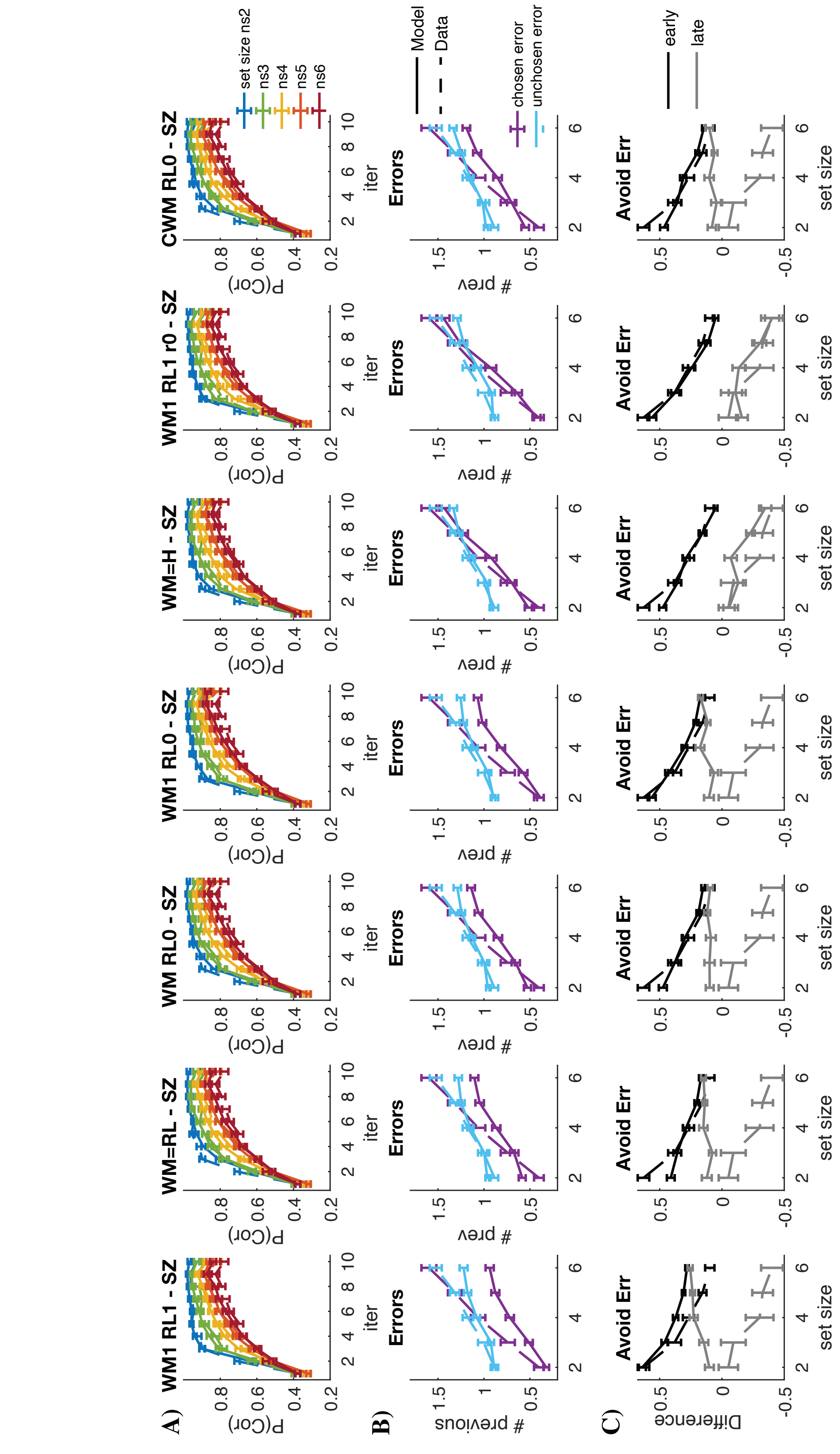
A)
B)
C)
WM1 RL1 - SZ
WM=RL - SZ
WM RL0 - SZ
WM1 RL0 - SZ
WM=H - SZ
WM1 RL1 r0 - SZ
CWM RL0 - SZ
P(Cor)
iter
set size ns2
ns3
ns4
ns5
ns6
Errors
previous
prev
Model
Data
chosen error
unchosen error
Avoid Err
Difference
set size
early
late

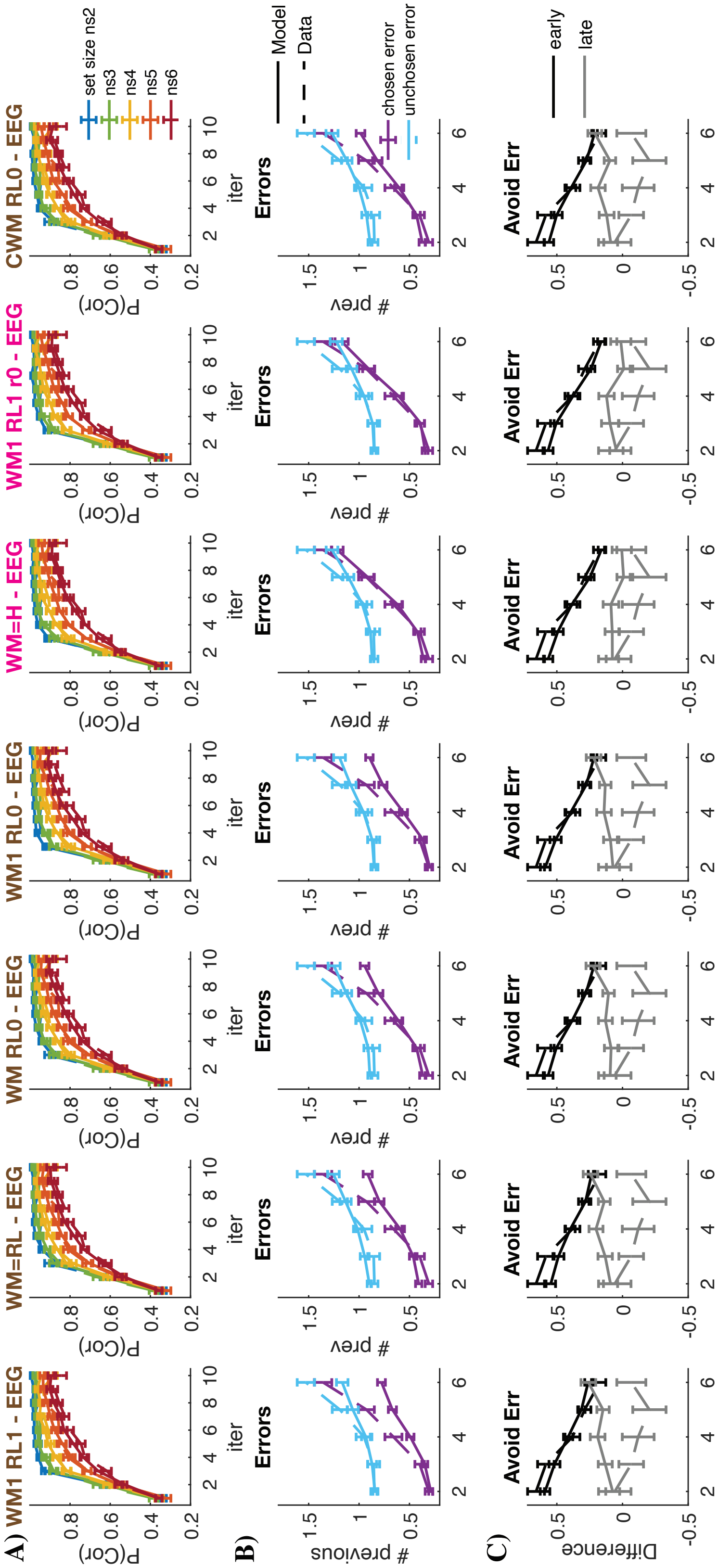
A)
B)
C)
WM1 RL1 - EEG
WM=RL - EEG
WM RL0 - EEG
WM1 RL0 - EEG
WM=H - EEG
WM1 RL1 r0 - EEG
CWM RL0 - EEG
set size ns2
ns3
ns4
ns5
ns6
P(Cor)
iter
Errors
previous
prev
Model
Data
chosen error
unchosen error
Avoid Err
Difference
early
late
set size

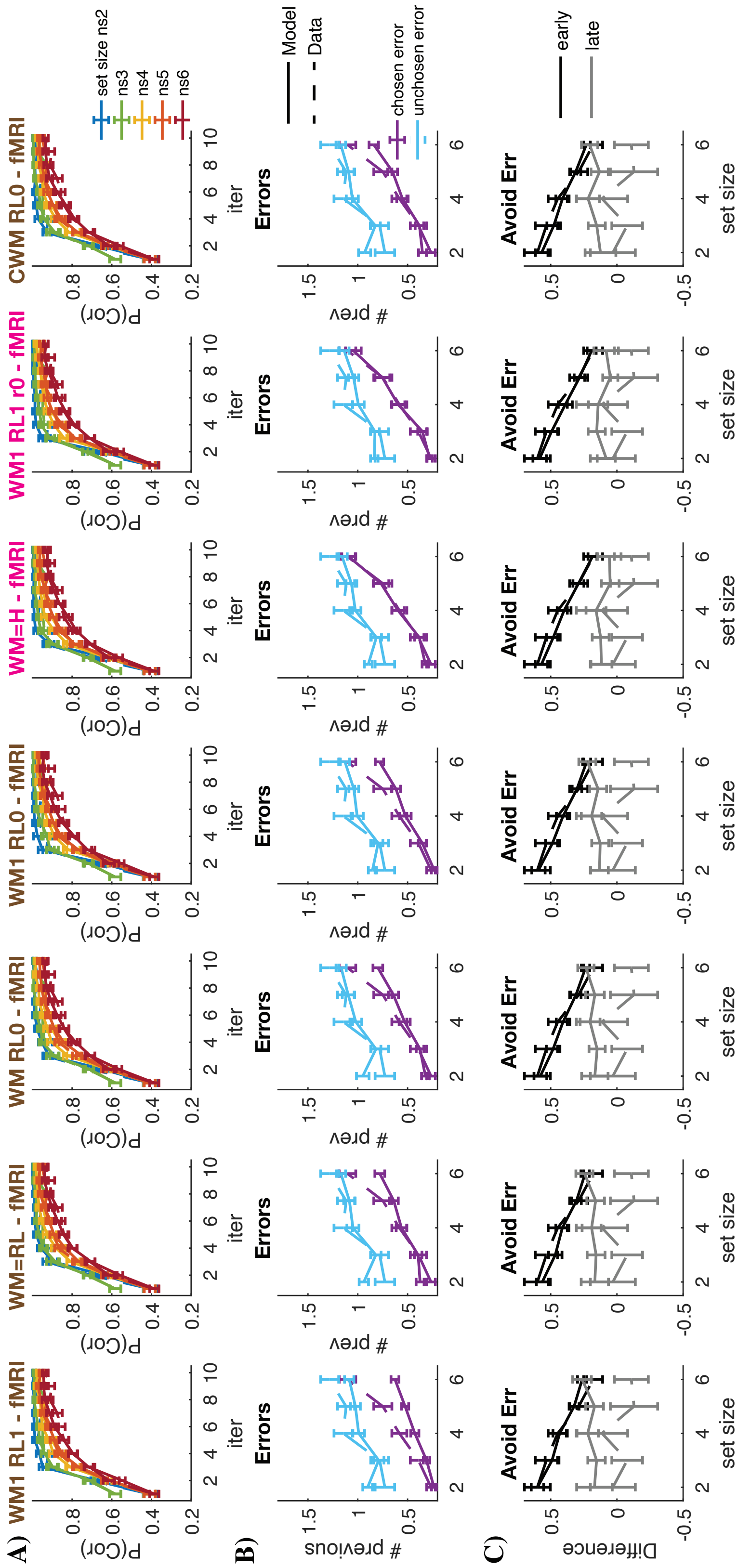
A)
B)
C)
WM1 RL1 - fMRI
WM=RL - fMRI
WM RL0 - fMRI
WM1 RL0 - fMRI
WM=H - fMRI
WM1 RL1 r0 - fMRI
CWM RL0 - fMRI
P(Cor)
iter
set size ns2
ns3
ns4
ns5
ns6
Errors
previous
prev
Model
Data
chosen error
unchosen error
Avoid Err
Difference
set size
early
late

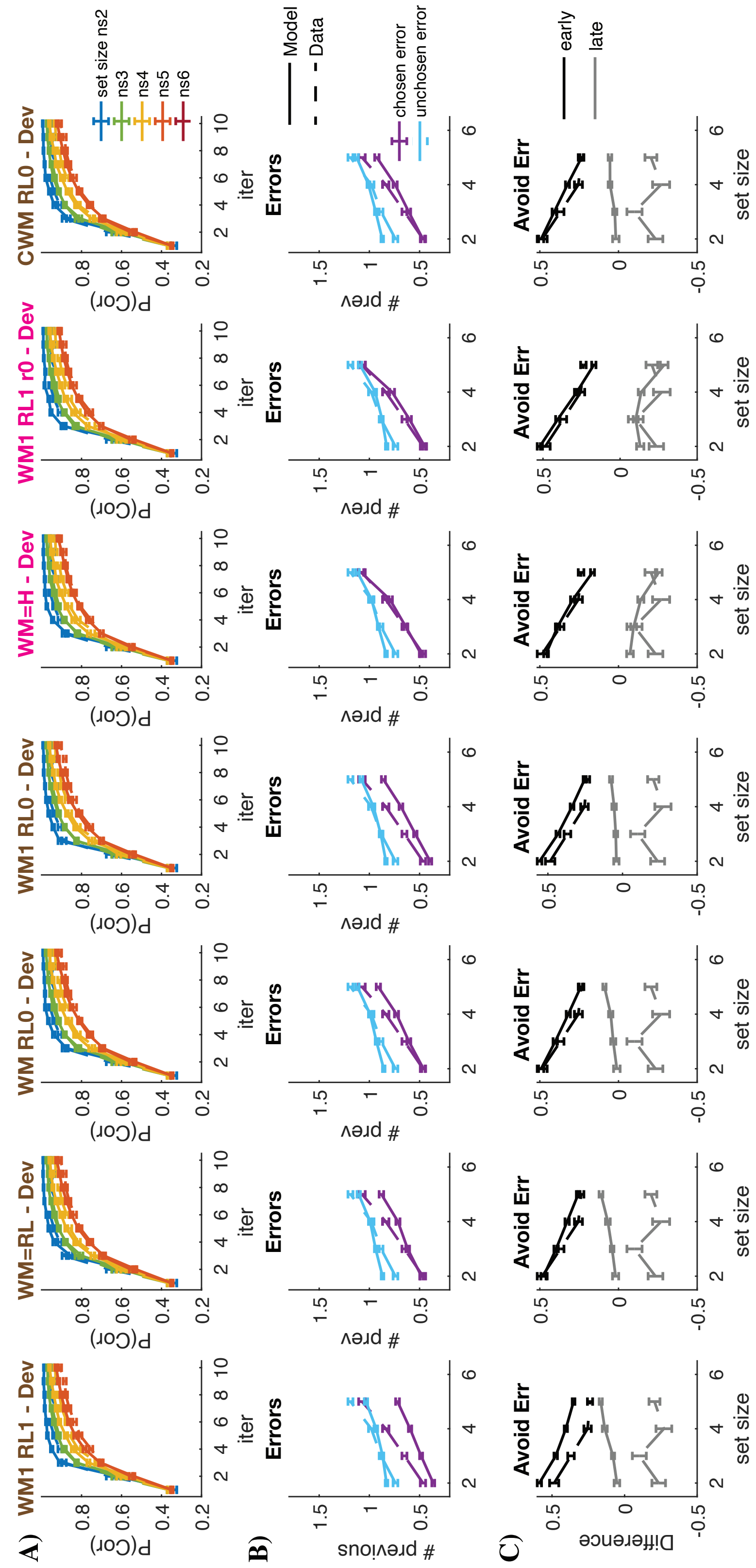
A)
B)
C)
WM1 RL1 - Dev
WM=RL - Dev
WM RL0 - Dev
WM1 RL0 - Dev
WM=H - Dev
WM1 RL1 r0 - Dev
CWM RL0 - Dev
P(Cor)
iter
Errors
previous
prev
Avoid Err
Difference
set size
set size ns2
ns3
ns4
ns5
ns6
Model
Data
chosen error
unchosen error
early
late

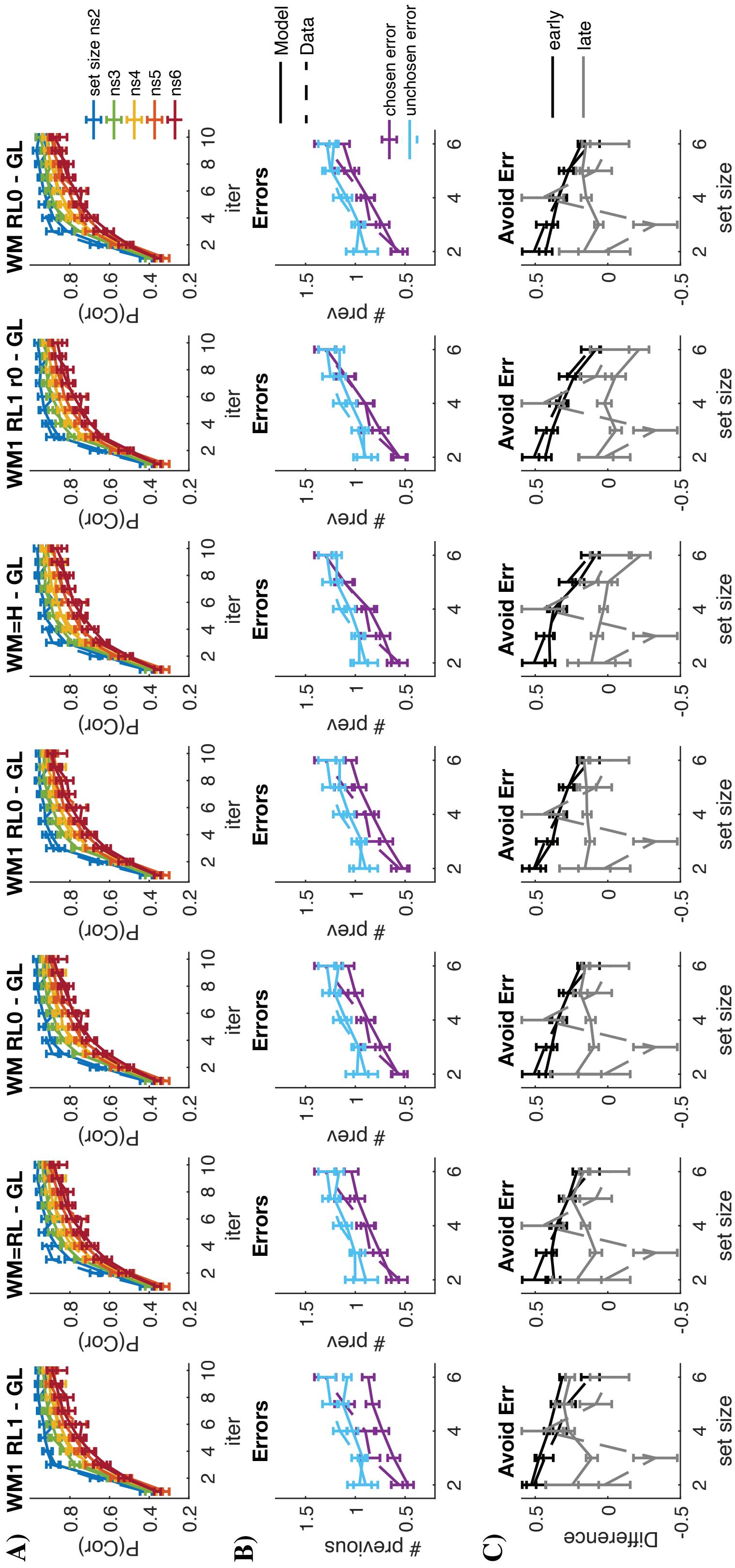
A)
B)
C)
WM1 RL1 - GL
WM=RL - GL
WM RL0 - GL
WM1 RL0 - GL
WM=H - GL
WM1 RL1 r0 - GL
WM RL0 - GL
P(Cor)
iter
Errors
previous
prev
Avoid Err
Difference
set size
set size ns2
ns3
ns4
ns5
ns6
Model
Data
chosen error
unchosen error
early
late

Figure S6

Validation figures for all 6 RLWM data sets. A) Learning curves per set size; B) chosen vs. unchosen errors as a function of set size; C) chosen-unchosen errors for early vs. late learning trials; See legend for main text fig. 2 for details. All data sets show the same results: that WMH models captured the learning curves and, most notably, error patterns better than RLWM models. Model WM1RL1r0 captured behavior as well as WM=H, (but fit quantitatively less well; see main text fig. 3). Figure S8 shows that the r_0 *parameter was typically fitted at a high value that would lead to positive reinforcement of negative outcomes, such that this model falls into the WMH category of agents (pink) for most subjects, rather than in the RLWM category (brown). For all plots, data is visualized as mean* $\pm$ *standard errors. The number of participants included are the same as in main text figure 1.*

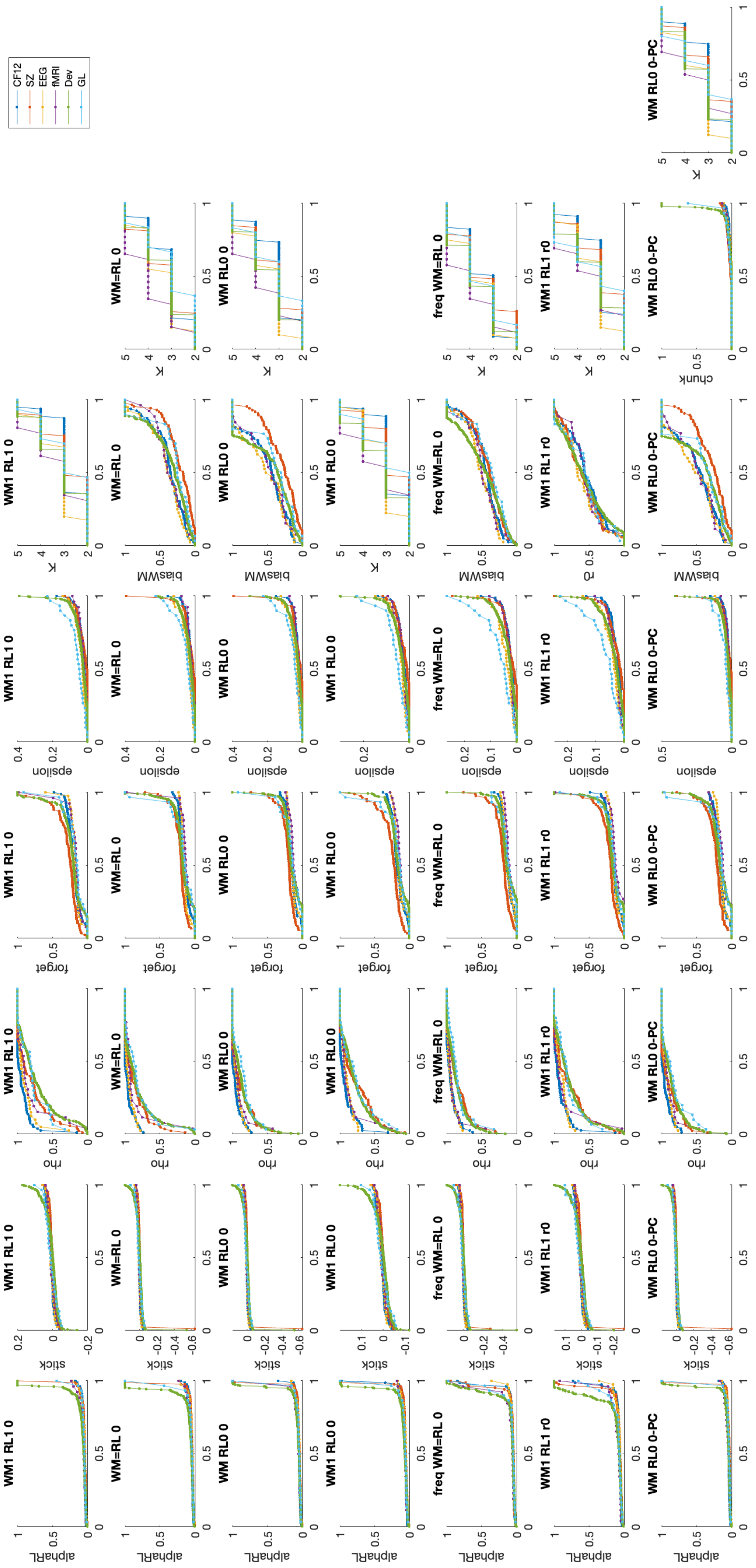
CF12
SZ
EEG
fMRI
Dev
GL
WM1 RL1 0
WM=RL 0
WM RL0 0
WM1 RL0 0
freq WM=RL 0
WM1 RL1 r0
WM RL0 0-PC
alphaRL
stick
rho
forget
epsilon
K
biasWM
r0
chunk

Figure S7

Best fit parameters for all 6 RLWM data sets, and the 7 models presented in main text figure 2 and in model validation above. Each row corresponds to a model (labeled in the title); each column to a model's parameter (y-axis). The x-axis represents sorted participants, with sorted order scaled to 0-1 to compare across group sizes. These curves can be interpreted as inverse cumulative distribution functions for parameters. Each data-set is coded as a different color (see legend in top-right corner). This figure shows that parameter values are broadly consistent across data sets, and very consistent across model variants.

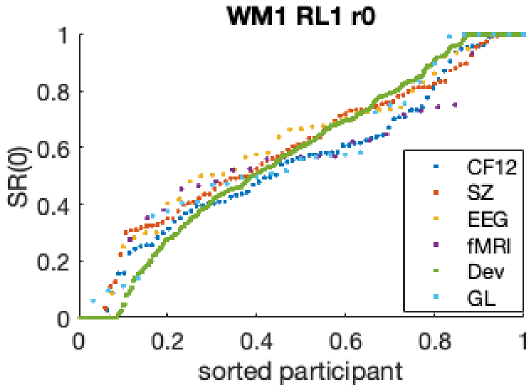

Figure S8

WM1 RL1 r0 model's fit r_0 *parameter distribution across data sets shows that a majority of participants are fit with a value higher than the initialization of* $Q_0 = 1/3$*, such that this model falls into the H family of the spectrum (making an error makes the error more likely to be repeated).*

RLWMP validation

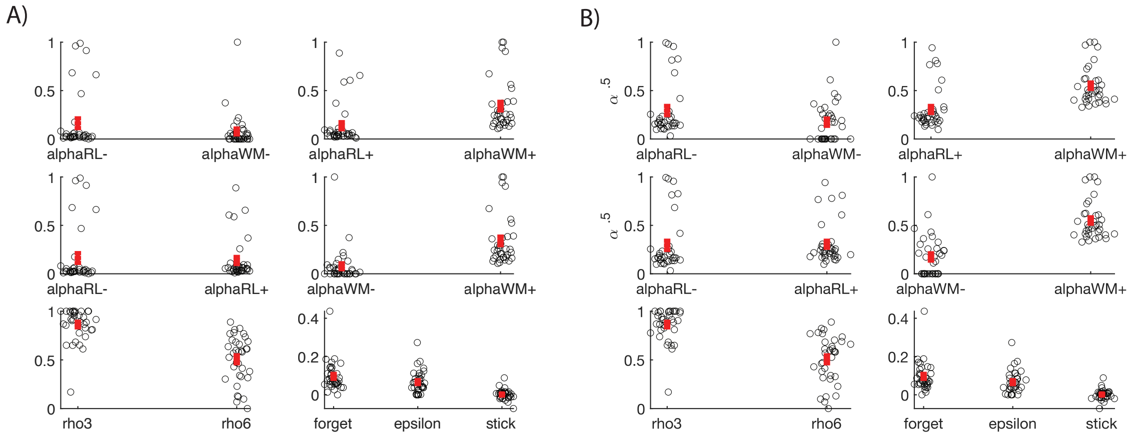

Figure S9

Fit parameters for the winning model in RLWM-P data set. A) Absolute parameters. B) for better visualization of the distribution of learning rates, we plot transformed parameters with $\sqrt{\alpha}$. *The WM positive learning rate is significantly higher than the RL one, highlighting the faster learning dynamic, as expected for a WM-based process. The weight to the WM process in ns3 is close to 1, as expected for WM use under low load. Red error bars indicate mean, SEM.*

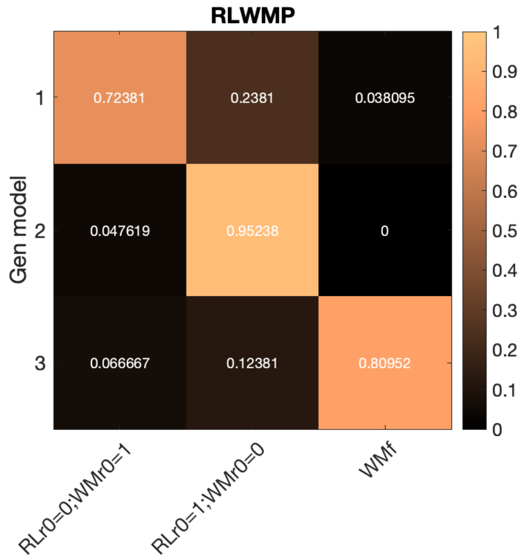

Figure S10

We use the same procedure as above to confirm model identifiability between the best model and competing ones.

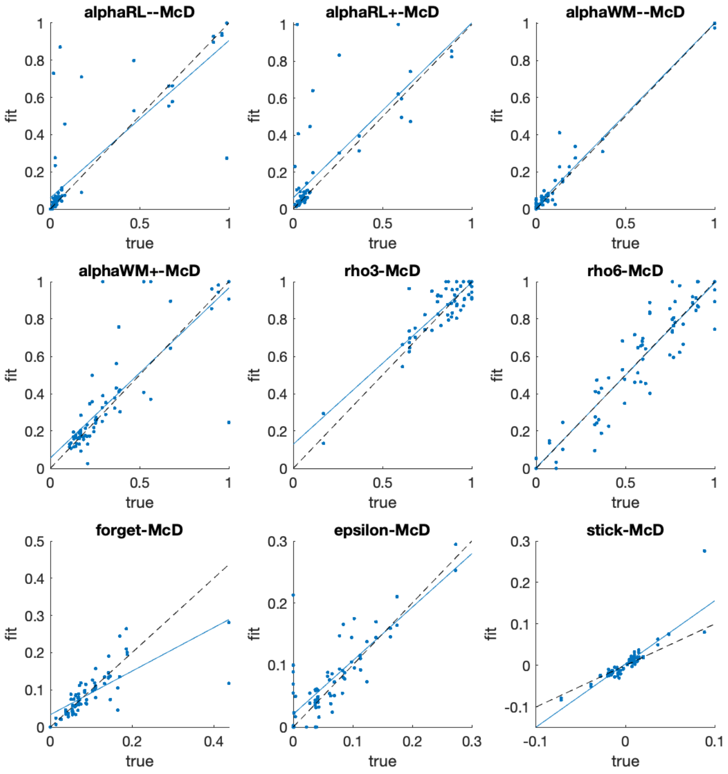

Figure S11

Parameters for the winning model in RLWM-P data set are identifiable, as shown by a generate and recover procedure.

Supplementary Discussion

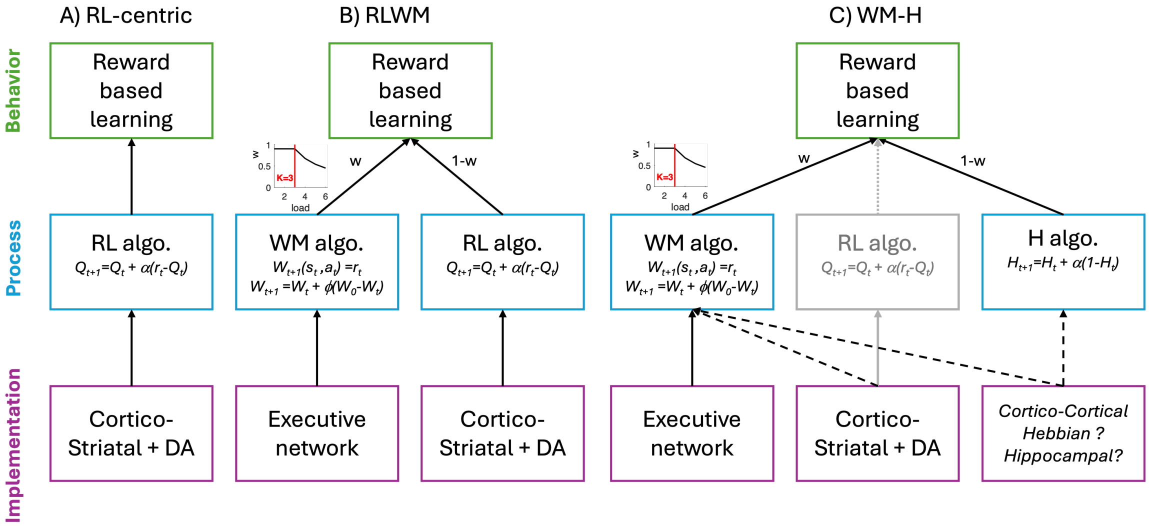

Figure S12

A)Standard RL-centric approaches to reward-based learning assume a close correspondance between the equations that govern behavior and their implementation in a cortico-striatal, dopamine-dependent (DA) network (Eckstein, Wilbrecht, & Collins, 2021) B) The RLWM framework factors out contributions of working memory, which are very high under low load, and decrease when load is under high capacity. C) Our results reveal no influence of RL-like computations on behavior (dotted line), but instead highlight an important H-agent contributing to reward-based learning. Underlying neural substrates are speculative (dashed lines). See methods for model equations and parameters. Note that, for simplicity, schematics B and C do not consider how processes may additionally interact (Collins, 2018; Collins, Albrecht, Waltz, Gold, & Frank, 2017; Rac-Lubashevsky, Cremer, Collins, Frank, & Schwabe, 2023).

Supplementary References

References

Collins, A. G. (2018). Learning structures through reinforcement. In *Goal-directed decision making* (pp. 105–123). Elsevier.

Collins, A. G., Albrecht, M. A., Waltz, J. A., Gold, J. M., & Frank, M. J. (2017). Interactions among working memory, reinforcement learning, and effort in value-based choice: A new paradigm and selective deficits in schizophrenia. *Biological psychiatry*, *82*(6), 431–439.

Eckstein, M. K., Wilbrecht, L., & Collins, A. G. (2021). What do reinforcement learning models measure? interpreting model parameters in cognition and neuroscience. *Current opinion in behavioral sciences*, *41*, 128–137.

Rac-Lubashevsky, R., Cremer, A., Collins, A. G., Frank, M. J., & Schwabe, L. (2023). Neural index of reinforcement learning predicts improved stimulus–response retention under high working memory load. *Journal of Neuroscience*, *43*(17), 3131–3143.
